# A Fully Collaborative, Noteless Electronic Medical Record Designed to Minimize Information Chaos: Software Design and Feasibility Study

**DOI:** 10.2196/23789

**Published:** 2021-11-09

**Authors:** Jackson Steinkamp, Abhinav Sharma, Wasif Bala, Jacob J Kantrowitz

**Affiliations:** 1 Hospital of the University of Pennsylvania Philadelphia, PA United States; 2 Sunnybrook Health Sciences Centre University of Toronto Toronto, ON Canada; 3 Emory University Hospital Atlanta, GA United States; 4 Carney Hospital Dorchester, MA United States

**Keywords:** electronic medical records, clinical notes, information chaos, information overload, clinician burnout, software design, problem-oriented medical record, medical records, electronic records, documentation, clinical, software

## Abstract

**Background:**

Clinicians spend large amounts of their workday using electronic medical records (EMRs). Poorly designed documentation systems contribute to the proliferation of out-of-date information, increased time spent on medical records, clinician burnout, and medical errors. Beyond software interfaces, examining the underlying paradigms and organizational structures for clinical information may provide insights into ways to improve documentation systems. In particular, our attachment to the *note* as the major organizational unit for storing unstructured medical data may be a cause of many of the problems with modern clinical documentation. Notes, as currently understood, systematically incentivize information duplication and information scattering, both within a single clinician’s notes over time and across multiple clinicians’ notes. Therefore, it is worthwhile to explore alternative paradigms for unstructured data organization.

**Objective:**

The aim of this study is to demonstrate the feasibility of building an EMR that does not use notes as the core organizational unit for unstructured data and which is designed specifically to disincentivize information duplication and information scattering.

**Methods:**

We used specific design principles to minimize the incentive for users to duplicate and scatter information. By default, the majority of a patient’s medical history remains the same over time, so users should not have to redocument that information. Clinicians on different teams or services mostly share the same medical information, so all data should be collaboratively shared across teams and services (while still allowing for disagreement and nuance). In all cases where a clinician must state that information has remained the same, they should be able to *attest* to the information without redocumenting it. We designed and built a web-based EMR based on these design principles.

**Results:**

We built a medical documentation system that does not use notes and instead treats the chart as a single, dynamically updating, and fully collaborative workspace. All information is organized by clinical topic or problem. Version history functionality is used to enable granular tracking of changes over time. Our system is highly customizable to individual workflows and enables each individual user to decide which data should be structured and which should be unstructured, enabling individuals to leverage the advantages of structured templating and clinical decision support as desired without requiring programming knowledge. The system is designed to facilitate real-time, fully collaborative documentation and communication among multiple clinicians.

**Conclusions:**

We demonstrated the feasibility of building a non–note-based, fully collaborative EMR system. Our attachment to the *note* as the only possible atomic unit of unstructured medical data should be reevaluated, and alternative models should be considered.

## Introduction

### Inefficient Electronic Medical Records: Why It Matters

Clinicians spend much of their workday using electronic medical records (EMRs), both getting data *in*—entering new information about patients—and getting data *out*—searching for and summarizing past information. Some studies estimate that ambulatory physicians spend approximately 50% of their work time navigating, searching, and entering information into the EMR [1]. Therefore, optimizing clinical information management systems is crucial for clinician efficiency, satisfaction, and high-quality patient care. Poorly designed information management systems lead to the proliferation of out-of-date or incorrect information [2-4], increased time spent searching medical charts [1,5,6], medical errors [3,4], and clinician burnout [5,7-10], while limiting the effective use of EMR data for individual or population-level applications.

Given the scope and impact of the problem, we must critically evaluate the core conceptual assumptions that underpin our current practices. For instance, are *notes* the best organizational unit for unstructured clinical data? Should clinicians in different teams always document in separate locations? Which data should be recorded and stored in a *structured* fashion, and which in an *unstructured* fashion? Historically, these questions have been informed less by principled usability concerns and more by regulatory requirements, vendor incentives, and holdover intuitions from the era of paper records, which have fundamentally different capabilities and limitations than electronic systems. A robust field of EMR usability studies would enable empirical examination of our intuitions regarding the optimal management of clinical data using controlled studies [11-16].

### What Creates Inefficient EMRs: Chaos and Complexity

Patient care is exceedingly complex, and the practice of medicine has increasingly recognized this fact [17-19]. Medicine has heightened its focus on holistic care, population health, and the behavioral and socioeconomic causes of medical diseases. However, the EMR systems that have emerged in the past 20 years are inappropriately suited to clinicians’ mental models and the realities of modern medical practice [20].

Within the EMR, clinical data are stored in multiple formats: unstructured clinical notes, semistructured reports, and structured lists of laboratory values, medications, problems, and allergies. Beasley et al [2] defined a framework, information chaos, for analyzing the information problems that arise in modern electronic charts and plague physicians. A physician’s ability to diagnose and treat heavily relies on the interpretation of information, much of which is stored within the EMR, such as the subjective history, physical examination, vital signs, laboratories, and imaging findings. Information chaos is a theory that highlights the spectrum of difficulties incurred in processing this large volume of information, which hinder timely access to this information. In addition to producing delays in care, these obstacles result in a substantial but nonessential expenditure of cognitive effort on the part of clinicians, ultimately contributing to higher rates of clinician burnout and medical error. The theory delineates five major hazards of information chaos: information overload, information underload, information scatter, conflicting information, and erroneous information. These hazards reinforce each other in overt and subtle ways (see Multimedia Appendix 1 for additional details). A user-friendly EMR should incentivize behaviors that minimize information chaos to facilitate quick and accurate clinical decisions and summarization. In contrast, poorly designed systems and paradigms *increase* the likelihood of behaviors that lead to redundant, conflicting, scattered, and erroneous information, inhibiting excellent care.

Although EMR system designs exacerbate information chaos, a clearly delineated educational framework for teaching and assessing physician-learner EMR skills could ameliorate some of these issues. The reporter, interpreter, manager, and educator (RIME) framework provides an educational framework that could be readily extended to EMR competencies, as outlined by Stephens et al [21] (RIME/EMR scheme). They argue that the Accreditation Council for Graduate Medical Education core competencies are applicable to EMR skill development and clinical practice and that the importance of information technology in facilitating lifelong physician learning necessitates an examination and careful planning of educational strategies to develop effective EMR users. As physicians develop from reporters to educators clinically, their abilities to find relevant data, order appropriate tests, and document thoroughly and accurately within the electronic systems must improve to match. However, the lack of intelligent organization of the data, unnecessarily complicated ordering systems, and paper-era documentation tools impede the advancement of these skills. A user-friendly EMR, designed through the lens of the RIME/EMR scheme, could provide easier reporting tools; guide rails for learning to interpret, integrate, and plan based on clinical data; and decision support based on the best, up-to-date evidence in nearly real time. Such a system is reminiscent of Weed’s [22] proposed system that *guides and teaches*.

### What Creates Inefficient EMRs: Dealing With Structured and Unstructured Data

In modern EMR systems, limited classes of structured data are recorded and viewed within *non-note* interfaces. The historical distinction between which data are structured and unstructured is largely arbitrary and a historical consequence of the "Meaningful use" initiative and other regulatory initiatives. Storing information in a structured form facilitates data-specific interfaces (eg, medication reconciliation interfaces), clinical decision support (CDS) tools, and automated population-level analyses. However, entering data in a structured format is more onerous for the clinician than the large, unstructured, free-text blocks such as those found in notes, particularly if the interface requires interruption of cognitive flow or multiple clicks to navigate between structured data entry points. Furthermore, individual clinicians are unable to define their own structured data elements to improve their workflows. We should question the traditional distinctions between structured and unstructured data. Specifically, the principle of designing to minimize information chaos can be used to guide a more principled understanding of structured data—historical precedent alone should not be the guide for system design.

### How the Current Idea of the Note Contributes to EMR Inefficiencies

Bundles, defined by Gorman [23] as “organized, highly selective collections of information,” are used extensively by clinicians to manage information in health care settings characterized by uncertainty, frequent interruptions, and grave outcomes. In these settings, time and attention are limited, and interdisciplinary care is essential. Effective design and selection of information bundles is critical to effectively communicate, filter, and act on medical information.

Currently, the most common EMR paradigm for documenting unstructured information uses bundles called *notes*. A note is simply a bundle of text that contains a group of related but distinct clinical observations and decisions. Notes generally organize unstructured information in three major ways: (1) by time slice (each note focuses on events and reasoning from a discrete, limited span of time), (2) by clinical thread (each note focuses on particular clinical topics and omits others), and (3) by responsibility (each note’s writer is responsible for the entirety of the note’s content). These 3 principles of unstructured data organization are sensible (Table 1); however, a note, as currently conceived, is a poor choice of information bundle that directly creates information chaos.

Note-based organization causes information chaos at two major levels: (1) at the level of the single clinician duplicating information in multiple notes over time and (2) at the level of multiple clinicians or teams duplicating information in independent clinical threads.

**Table 1:** The 3 major ways that notes organize unstructured information into discrete bundles.

	Organizational paradigm		
	Time slice	Thread	Responsibility
Description	Each note holds information from a particular time slice of a patient’s history.	Different sets of notes focus on sets of particular clinical topics (a thread) and omit or deemphasize others.	The writer of each note is responsible for and simultaneously attests to all information within it.
Examples	Each progress note in a set of hospital notes holds information about a particular 24-hour period; each outpatient visit holds information about the period between the previous visit and that visit.	A thread of outpatient progress notes focuses on the management of chronic problems; a thread of nursing notes in a hospitalization focuses on nursing care issues; and a thread of cardiology consult notes focuses on the patient’s cardiology history, exam, diagnoses, and treatments.	Attending physicians and trainees may write separate notes or note segments containing the same information.
Utility	Enables sorting and filtering information by time	Enables limited filtering by subject matter (for consult services, social work notes, and nursing notes) Enables entirely independent documentation by clinicians in different threads	Enables clear assignment of responsibility for a particular set of facts and reasoning.
Source of information overload or duplication	Most of a patient’s medical information remains the same from time t to time t+1; however, it is often redocumented or copy-pasted forward from a previous note.	Most of a patient’s medical information is shared among different clinical teams or threads; however, it is redocumented or copy-pasted in multiple independent note threads.	Information may be redocumented multiple times or by multiple clinicians rather than simply *attesting* to one’s agreement with a colleague.
Source of information scatter	Difficult to track the course of a chronic problem over time (it either requires navigation among many notes or accumulative charting, where each note continues to grow in size and hold the entire history of the patient) Difficult to document small single-fact updates without making an entirely new note	Difficult to see where clinicians disagree on a particular issue (history element and examination finding), as it requires navigation among multiple notes	N/A^a^

^a^N/A: not applicable.

The first problem may be caused by 2 rival conceptions about what a note as time slice is supposed to be. On the one hand, a note can be seen as a *bundle of new updates*—only holding the information that has *changed* since the previous note. This minimizes information duplication but increases information scatter; tracking a patient’s problem over time requires navigating across many notes. On the other hand, a note can be seen as a complete *current state of the patient*. In this view, anyone reading only a single note has a complete picture of the relevant information at that time. This practice minimizes information scatter but leads to charts bloated with duplicate information, making it difficult for a later reader to identify what has changed from time *t* to *t+1*, and makes it onerous to expunge errors from the chart. This conception of the note forces us into a trade-off between information scatter and information overload. In addition, information is commonly duplicated across multiple note threads, although most of the patient’s clinical data are the same. The information that does differ is usually unintuitively scattered in different places, making it difficult to reconcile conflicts, thereby limiting the potential for meaningful collaboration within the EMR. In this study, we aim to develop a novel EMR system that uses a fully collaborative, dynamic, and problem-oriented approach to minimize information chaos within electronic patient charts. We aim to explore the pros and cons of a non-note paradigm in an attempt to expand the dialog around EMR usability.

## Methods

### Overview

We sought to design a system that breaks down the traditional barriers of structured and unstructured data based on the notion that the current organizational principle of *notes* is a major contributor to information chaos within the EMR. In particular, we believe that the rigid organization of unstructured data by *time slice, thread,* and *responsibility* leads to information overload and information scatter, which in turn may lead to wasted time and effort, medical errors, user dissatisfaction, and clinician burnout. We hypothesize that it is feasible to build a modern EMR that does not use notes as the major organizational units for unstructured data and allows clinicians the flexibility they require to perform an unpredictable job.

### Design Principle: Designing to Minimize Information Chaos

To prevent information overload and scatter, the core operating principle of a clinician using our EMR is as follows: redocumentation is to be fundamentally disincentivized at every aspect of the system’s design. Taken to its logical extreme, in an ideal system, a user should find it *absurd* or *unthinkable* to redocument the same information multiple times within the same patient chart. Details will be discussed in the *Results* section, but the key principles are listed below.

First, within an individual clinician or team’s *thread* of documentation over time, the default assumption is that information will remain the same over time; information that changes is the *exception.* This is a sensible assumption, given that a patient’s accumulated past medical history will rarely be changed once recorded, and many aspects of a patient’s current medical state will remain the same between 2 consecutive clinical notes. This means that our system’s interfaces should be designed around *small-scale changes of individual data elements* without requiring redocumentation of the remainder of the unchanged data—something not possible under a note framework, which usually treats the *note* as the atomic unit of writable and viewable information. In contrast, we identify *individual data elements* as the atomic unit of writable information rather than the *note*, which is, by definition, an aggregate of many data elements. This is the first intuition that leads us to reject the *note* as an information organization principle.

Second, clinicians on *different* teams or services (ie, those who would traditionally use different note *threads)* should not be required to maintain separate copies of the information that does not differ among teams or services. We would seek to reduce the number of distinct clinical threads and incentivize the shared maintenance of an entirely *collaborative* chart wherever possible. This has numerous benefits, including reducing information overload, information scatter, and error propagation while facilitating more meaningful collaboration within the EMR. This requires dissolving the sharp boundary between notes written by different teams in favor of a default collaborative workspace. Although this may initially worry some readers, we will explain in detail how we deal with the concerns around responsibility tracking in the *Results* section. We believe that the benefits significantly outweigh the costs.

Third, wherever it needs to be recorded that information *has not changed* since the last documentation event, the fundamental mode of doing so will be *attestation,* not *redocumentation.* A clinician can attest to the current state of the information they are responsible for without typing it out again (or even copy-pasting it). This saves time and effort while reducing information overload. This principle also requires us to reject the *note* as the atomic information unit in favor of something more like a dynamic *living workspace*.

### Design Principle: Baked-In CDS

Another key design principle, which our system shares with the traditional problem-oriented medical record [22], is the notion that medical records should *guide and teach*—that is, facilitate learning and CDS at the problem level. For instance, if a clinician indicates that a patient has congestive heart failure (CHF), the system should present the clinician with a framework for how to think about that problem, including relevant data to be collected, active diagnostic possibilities, actions to be considered (including diagnostic tests, therapeutic orders, consults, and dispositional concerns), and links to external information resources. These frameworks should be informed by other information already captured in the chart—for instance, if a cardiac history was already captured in the context of a coronary artery disease workup, that information should be viewable by default from within the CHF problem, removing the need to search for that information (and, if unchanged, the need to *redocument* it).

In this way, CDS is *baked-in* at the point of diagnosis and patient conceptualization, not merely at the point of structured data entry (eg, medication orders). A good documentation system serves as a *mental scratchpad*, which mirrors clinician cognition while providing suggestions and support to overtaxed human brains [24,25]. Such systems would not only help trainees learn how to think about particular problems but also facilitate the incorporation of the latest treatment guidelines and research studies by clinicians at all levels of training. Given the large volumes of information required for modern medical practice, such a system would likely serve an important *checklist* role, freeing physicians to focus on more uniquely human cognitive and emotional concerns. A proper system would allow for sensible defaults at the institution level but would *always* enable clinicians to build their own custom individual templates for their own practices. Textual templates in existing note-based EMRs enable some of these capabilities but do not have the kind of granularity or conditional logic necessary to achieve the full potential of such a system, largely because of the constraints of a note-based organization.

Then, a necessary component of a non–note-based system is an engine that enables clinicians to build their own granular problem-based CDS and workflows. We describe our implementation of such a system in the *Results* section.

### Software

Our system is a web-based EMR system. All the server-side code was written using the Python language with the *flask* web framework. On the back end, we used the MongoDB database to store app data. The front end is written in JavaScript using the Vue framework. The SocketIO framework was used to facilitate real-time multiuser collaboration and push notification functionality. When an update to a patient’s chart is made, the server notifies all other active users of the change, enabling real-time collaboration. For further details on the implementation of real-time collaboration, see Multimedia Appendix 2 [26]. The software was developed iteratively with multiple rounds of idea generation, user testing, and feature refinement.

## Results

### Overview

**Figure 1:**
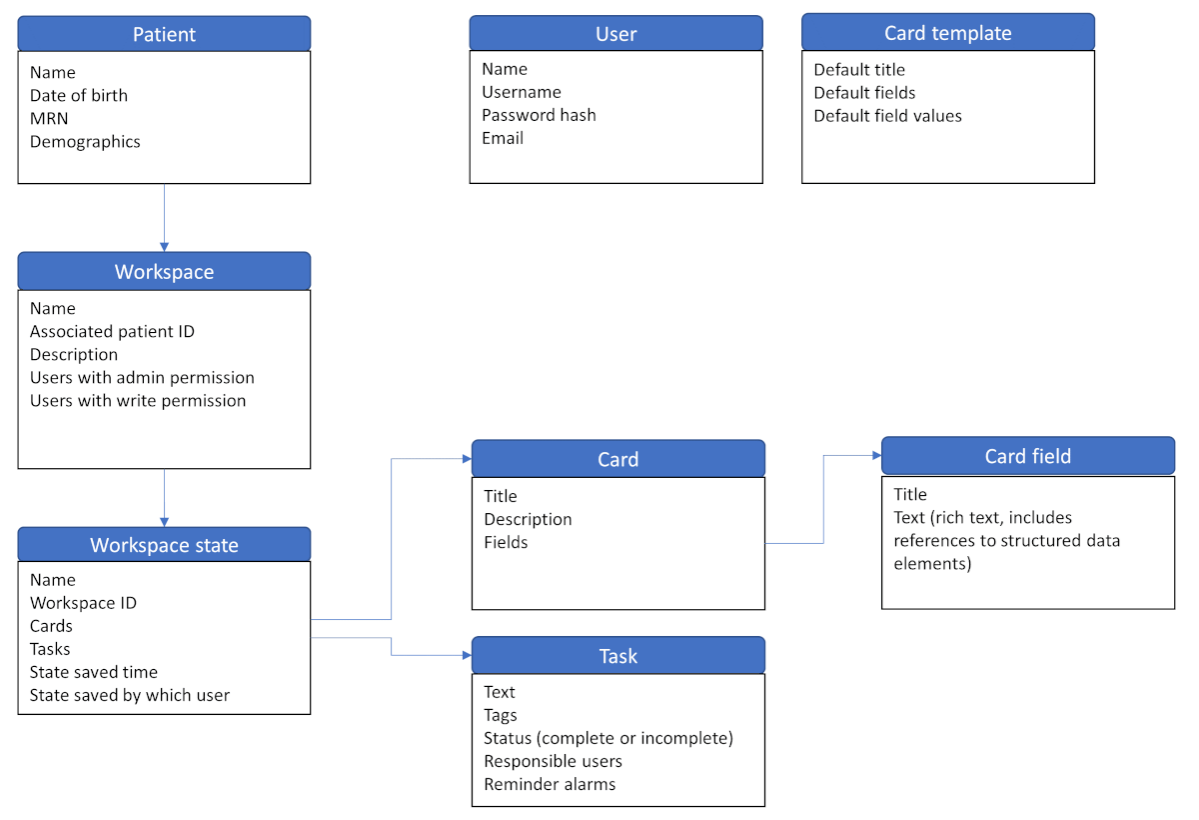
Database diagram showing the organization of the major conceptual entities in the system. MRN: Medical Record Number.

Below, we describe the major components of the software and their implementation. Each of these key features is designed to decrease information chaos—particularly the duplication and scatter of related information in disparate locations. Figure 1 shows the overall relationships between the different conceptual entities and data structures in the MongoDB database.

### Cards

**Figure 2:**
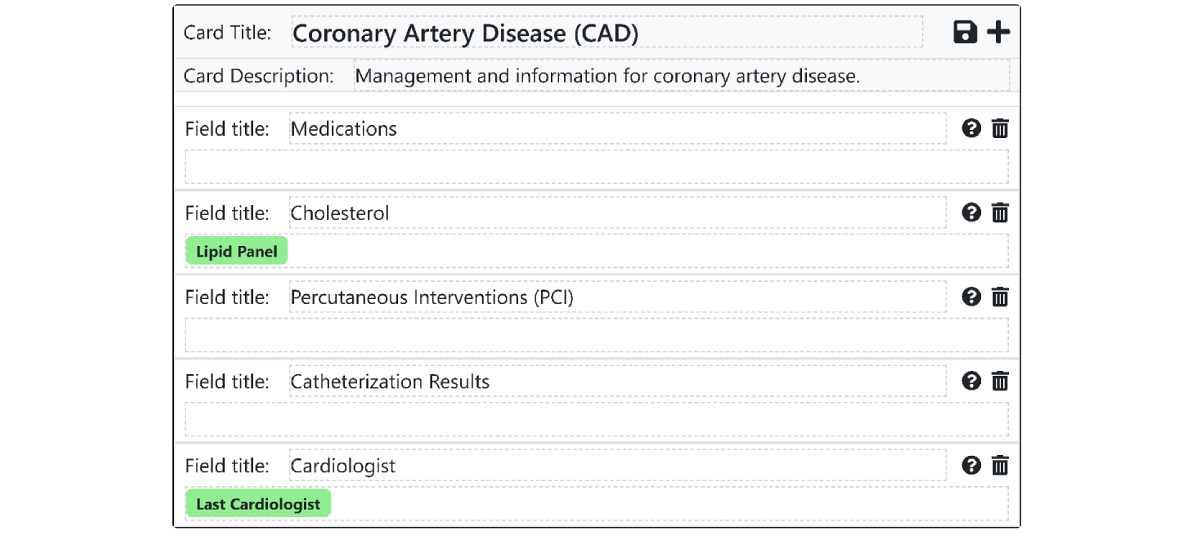
An example of a problem card template comprising a title, description, and list of free text data fields. Users build these problem card templates and instantiate them for a particular patient. The green bars represent structured data, which will automatically be pulled into the card when an instance of this card template is created. The cards add flexible, customizable structure to the topic-based documentation process.

**Figure 3:**
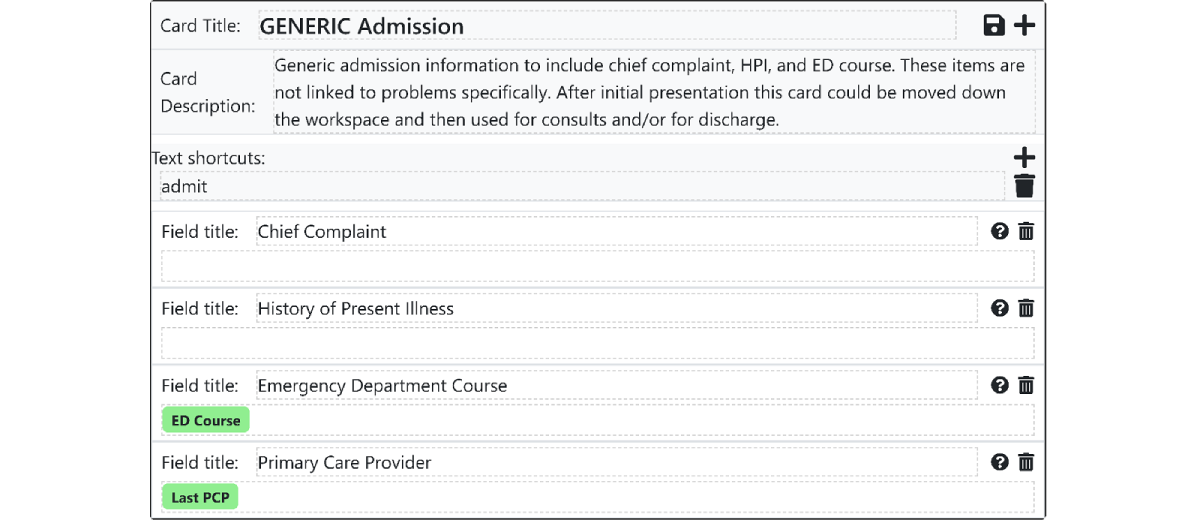
An example of a card that does not map to a medical problem. The flexible structure of custom cards enables users to build their cards into the workflow. Note that ED course and PCP are structured data elements built by the user, which can be pulled automatically into multiple cards while retaining a single source of truth. ED: emergency department, HPI: history of present illness, PCP: primary care provider.

Under a *note*-*based* paradigm, all information covering a particular time slice of a patient’s medical life (eg, a single day of hospitalization) must be stored together in one note, regardless of content. As discussed above, this organizational scheme forces users to write notes that are overloaded with information or that are underloaded, with information scattered across many notes. Instead of the note as time slice approach, our system takes inspiration from work on the problem-oriented medical record [22] and organizes information by topic to facilitate charts that *teach and guide*. In our software, each topic is represented by a *card*, which holds a set of information related to that topic. Each card consists of a title and multiple *fields* that provide additional structure within the topic (Figure 2). Cards are frequently used to capture individual medical problems; for instance, a CHF exacerbation card might hold fields representing the patient’s history of present illness, relevant physical exam findings, outpatient clinicians, laboratory and imaging results, and treatment orders. However, cards are designed to be flexible enough to capture topics that do not neatly map to medical problems, such as "demographic data", "cardiology consult recommendations", "list of outpatient clinicians", "nursing comments", or "overnight updates" (Figure 3). This enables institutions, teams, or individual clinicians to develop cards customized to their own individual workflows. The fields are, by default, all free-text, retaining all the advantages of textual data while adding a small amount of flexible structure. Each card field’s data are stored and updated separately in the database rather than every topic being stored together in a single block of free text. This enables granular viewing of individual cards or fields and their changes over time, in the case where a clinician wants to quickly see the history of a patient’s CHF.

However, the default chart view shows all currently active cards for a patient regardless of how recently each piece of information has changed. This is sensible, as different medical information update at different frequencies. Even for the same problem, a patient’s acute status may change daily, whereas their outpatient medications may change monthly, their providers change still less often, and their demographics may never change. Therefore, clinicians can get a quick understanding of the current state of the patient without having to navigate back through numerous notes to find updates that occurred at different times. Our system shares these advantages with other forms of *problem-based* or *topic-based* charting.

By using cards as the basic organizational principle, we escape the trade-off between information overload and information scatter. Our system facilitates granular updates to individual topics without requiring large-scale redocumentation and information overload. For instance, if everything about a patient’s CHF has remained the same from day 1 to day 2, the card does not need to be modified at all. Instead, the responsible clinician simply attests that no information has changed, which saves time and does not create a duplicate copy of the same data (as is the case with notes). Updates that consist of only 1 or 2 factual updates to the chart (ie, a telephone call to refill medication) require clinicians to only update a single card field. Unlike a note-based organization, which would scatter topically related information across multiple notes, the relevant data will be stored together regardless of when it was documented, enabling easier viewing of longitudinal changes. Therefore, the card-based system cuts down on both information overload and information scatter. This structure also enables readers (or automated systems) to quickly identify which cards and fields have changed from update to update and which have remained the same. We will discuss this further in the *Version History* section.

The cards also facilitate real-time collaboration. Multiple clinicians can work on individual cards or even different fields within the same card at the same time, reducing the incentive for individuals on the same team to create separate notes. Furthermore, card-based organization can reduce the number of separate clinical threads; ideally, inpatient consultants would be responsible for individual cards or fields within a general inpatient workspace, obviating the need for the consultant to redocument large amounts of the patient’s history of present illness medication list, etc. Real-time collaboration is facilitated using web sockets, which allow for two-way communication between client and server; when a client makes any update to a card within a patient workspace, the server will push the update via the web socket, enabling other users to see the changes immediately. This facilitates the reconceptualization of the chart as a *dynamic living workspace*.

### Structured Data

**Figure 4:**
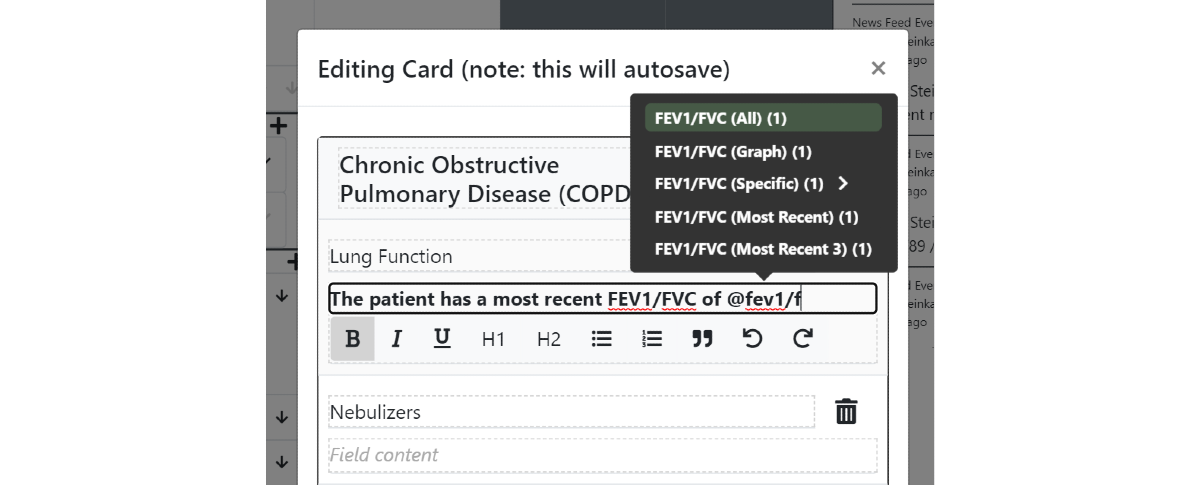
Use of the structured data display operator. Typing @ in a card field will create a menu for different structured data elements, which can be pulled into the text of a card field, including the ability to create graphs and lists. The user is free to define and customize their own structured data elements.

**Figure 5:**
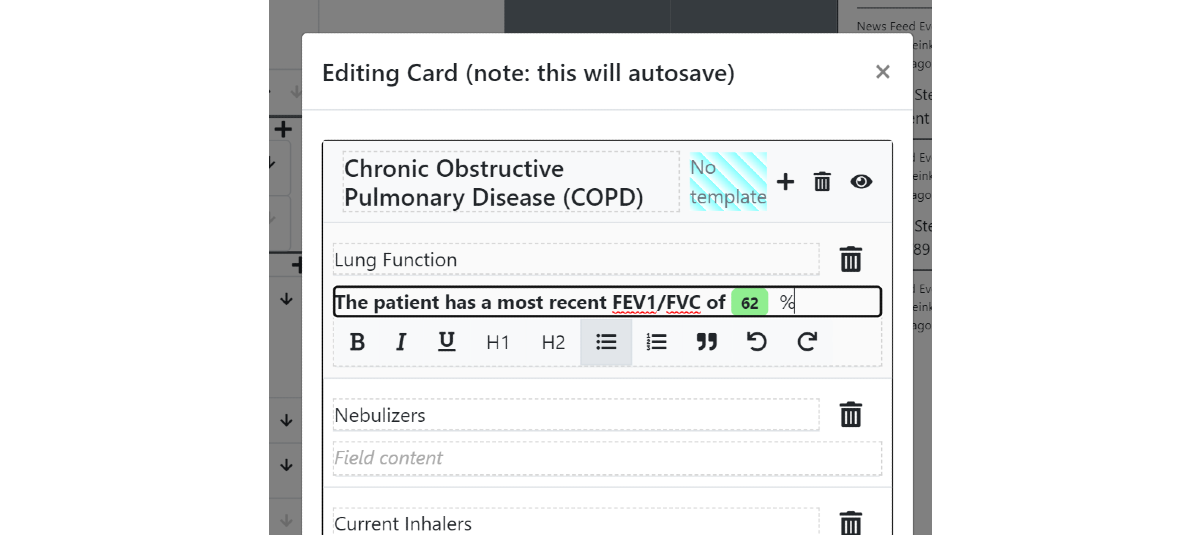
Clicking one of the menu options will pull data from the shared structured data pool. It is highlighted in green to indicate that it is an updatable structured data element, not a raw text.

**Figure 6:**
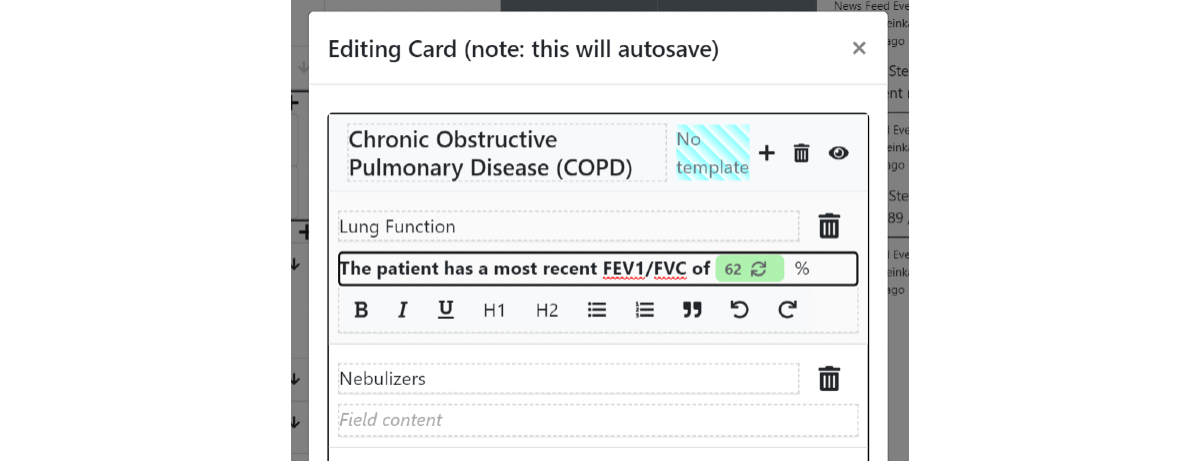
The refresh icon appears in a card if the underlying structured data element has changed. If the user wants to maintain a particular structured data value from a particular time (eg, the sodium on admission), the refresh icon will only appear if that value is updated. When a user wants to track the most recent values of a structured data field (eg, the most recent ejection fraction at the time of viewing that card), the refresh icon will appear if there are any more recent values for the same structured data element (eg, a more recent forced vital capacity than the one currently displayed).

**Figure 7:**
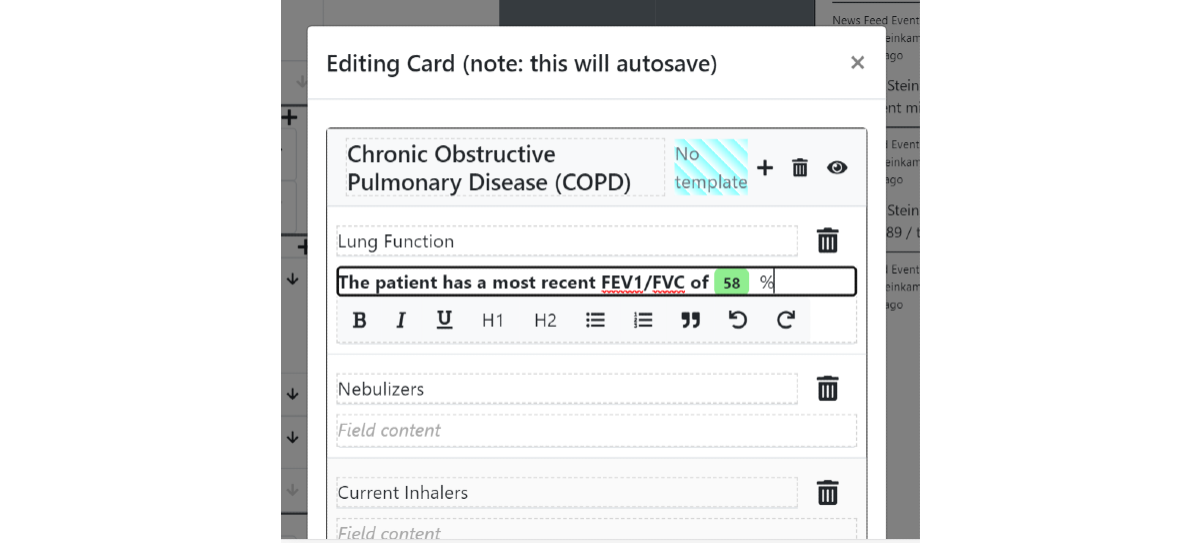
When the refresh icon is clicked, the data element in the card field updates to reflect the changes without requiring the user to view the structured data element.

Structured data elements can be pulled into unstructured documentation using an *@* operator inside any of the free text card fields, akin to a mention or hashtag system in other software—we call this the *structured data display operator* (Figure 4). For instance, if “@sodium” is typed inside a card field, a set of options related to the stored sodium laboratory values will appear. In its simplest form, a reference to the latest sodium value will be pulled into the text field (Figure 5) and displayed in a rich text format. Critically, this structured data value is not a disjoint piece of text but a reference that can be updated as new information arrives. If the user wants to track the latest value of sodium, and a new value of sodium is input into the system, the textual reference will display a *refresh* icon, suggesting that the displayed value is no longer the latest value (Figure 6). The user can choose whether they wish to make use of the refresh icon to update their textual documentation. This is designed to prevent the propagation of incorrect values because a clinician forgot to update the text—a scenario that has the potential for significant medical error. We allow each structured data element to be displayed in multiple formats—a "last value" option, an "all values" option, a "specific value" option (which does not change with time), and a *graph* option, which enables a real-time updating graph of the latest values of a particular structured data element (Figure 7), such as a laboratory value. Note that these data elements are situated within the free text card fields, removing the requirement to navigate with multiple clicks to separate *graph viewing* interfaces and showing topic-relevant information in the same place—preventing information scatter, underload, and lost time.

To facilitate flexible, customizable workflows, we take the widest possible view of what counts as structured data by enabling individual users to define and create their own structured data elements, as well as enter any structured data value themselves (eg, laboratory values obtained from outside organizations). This includes traditional structured data elements, such as laboratory results, but is also designed to work with other numeric values (ejection fraction, pack-years of tobacco use, and risk scores) in addition to string values and categorical values (code status and the patient’s current outpatient pulmonologist). This empowers individual clinicians to quickly improve their templating systems.

### Template Engine

**Figure 8:**
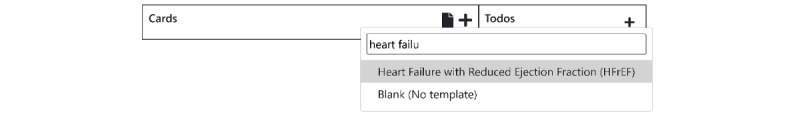
Card templating engine, enabling the design of templated problem-specific fields, which can pull in structured data (part 1).

**Figure 9:**
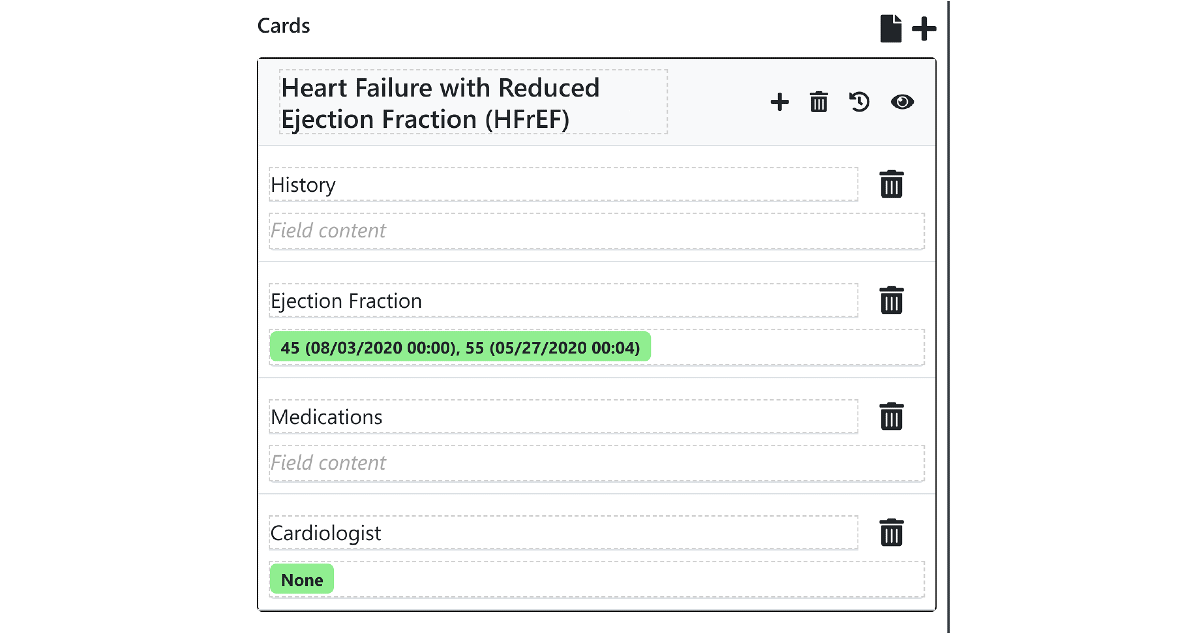
Card instantiated from template.

Most patient encounters involve a small set of common medical problems, suggesting that a significant benefit is to be gained from templated workflows. In current EMR systems, *note templates* are used to realize this benefit. We implemented a much more granular templating system at the level of *card templates* (Figure 8)*.* A card template consists of a set of prespecified fields with optional prespecified default text in each field. Inside a patient’s workspace, cards can be quickly instantiated from the templates (Figure 9). This default text can include structured data elements, enabling the clinician to (for instance) autopopulate a reference to the latest ejection fraction when a card is instantiated from a template. This is another advantage realized by a noteless EMR, and it is here where the potential of medical charts that *guide and teach* can begin to be realized. Trainees, in particular, may benefit from card templates, which provide a framework for thinking about a particular medical topic, including important diagnostic considerations, therapeutic decision points, and signs of patient status change, all of which can be included within a card template. Physicians play multiple roles, and these teaching medical charts can help us to be better clinicians, educators, and learners by providing perpetually up-to-date resources naturally within our existing workflows. Card templates can include embedded text from external resources such as guideline organizations and research studies, enabling not only clinical refreshers but also ongoing medical education.

Such templates have the potential to make clinicians at all levels more efficient and prevent medical errors caused by forgetfulness or outdated information. In this way, CDS is *built into* the information organizational framework in a way that provides guidance more than it aims to correct. Traditional CDS is typically based on structured data, meaning the support is necessarily given after data are created, in reaction to a particular pattern of data (eg, medication interactions, allergies, and laboratory values). On the other hand, by instantiating a card template with a particular structure, the card instantiation itself becomes a form of CDS.

In addition to a simple menu-based selection system for instantiating card templates, our system includes a separate interface that enables fast autopopulation of cards from free text, such as a patient *one-liner*. A one-liner is a single-sentence patient summary that lists, among other things, a patient’s relevant medical problems. Our system uses simple rules to identify which card templates should be created from a given block of text; however, more sophisticated natural language processing systems could be used to improve performance and handle more complicated linguistic phenomena. In the future, such a system could be used to import free text blocks directly from other systems that operate according to note-based organizational systems and quickly convert them to the more flexible card-based organization.

### Workspaces

Although our system is designed to cut down on the number of distinct clinical threads and the information overload that results from multiple clinicians or teams redocumenting the same information, we understand that there are potential use cases where clinicians should work in separate *workspaces.* Therefore, we allow for the creation of separate workspaces for the same patient, which recaptures the notion of separate *threads*. The system requires only a single click to navigate from one workspace to another in the same patient’s chart. Each workspace has specific user permissions: clinicians with write permission to a workspace can see the most up-to-date version of a workspace, even if it has not yet been attested. On the other hand, those with read permissions can only see the most recent *attested* version to prevent acting on incomplete information. For custom workflows, cards from one workspace can be *watched* from another. Watching a card creates a read-only reference to the original card in the new workspace and enables a clinician to view relevant information documented by someone else from another workspace. When the original card updates, the *watched* copy of the card will also update in real time. This is meant to replace the current behavior under a note-based system in which clinicians copy-paste information (eg, consult recommendations or a radiology report) from one text document into another to have all the information in one place. Although other design decisions of our system will ideally decrease the information scatter that motivates this behavior, card-watching is nevertheless present to mitigate the creation of duplicate *unlinked* data copies.

### Task Management

In many clinical care settings, relevant patient information is frequently passed between clinicians and staff through ancillary systems such as emails, pagers, SMS text messages, and separate handoffs. Our system includes a task management or *to-do list* tracking system as a core feature. Each workspace includes a dynamically editable, fully collaborative to-do list that enables task management for that patient. To-do lists can be assigned to particular users, can be associated with reminders accompanied by push notifications, and can be given custom user tags to enable filtering and searching functionality. These to-do lists can be viewed in aggregate across a group of patients, enabling efficient inpatient team and outpatient panel management.

### Real-Time Collaboration

Notes are not an effective way to communicate when there is some urgency to the task; immediate messaging systems are more appropriate for this use case. Therefore, our system includes a *chat* functionality as the primary mode of communication. The chat system enables direct messaging among clinical users in the system and includes a group chat functionality, which is similar to the chat functionality present in other EMR systems. In addition, in our system, each patient workspace is associated with its own additional chat, so short notes or messages related to a particular patient can be stored. However, unlike many other chat systems, ours is public and, thus, auditable. The public nature of the chat enables any system user, including physician colleagues or learners, nurses, and other staff, to understand the clinical decisions that have been made for their patients and to learn from these conversations, as opposed to these happening in closed-door sessions.

Within the workspaces, we include a real-time cursor tracking feature: a user in a patient chart can see which other users are editing that workspace and where their cursor is located to prevent multiple users from editing the same information at the same time and facilitate real-time collaboration (eg, by serving as a jumping-off point for a chat conversation between a primary clinician and a consultant).

Although some may be hesitant to treat the chart as a collaborative workspace, based on fears of medicolegal reprisal or insufficient assignment of responsibility to individuals, we believe our system is capable of handling the responsibility assignment issue at least as well as note-based systems. Each edit (addition or deletion of a card, editing of a card title or field text, etc) is tracked in a separate logging table, enabling fine-grained assignment of responsibility for individual edits to particular users. These logs also enable the ascription of responsibility for each piece of text in a workspace to particular users and particular time stamps. This feature can be helpful not only in cases of ascribing responsibility but also when a user wishes to orient themselves to a new chart and understand what information has been recently updated and what is in need of verification or updating.

### Version History

Under our system, the default assumption is that most information *will not change* from update *t* to *t+1.* This intuition conceives of the chart as a dynamic living workspace and facilitates an intuitive version history system akin to that found in modern word-processing and other collaborative software. Each patient workspace consists of a set of states representing how the workspace has been updated over time by any number of clinicians working collaboratively. In particular, each workspace contains a single *current* state, which reflects the latest view of the workspace, and any number of past states, which reflects how the workspace looked at particular points in the past. Each state consists of a set of cards, to-do tasks, and structured data elements, as mentioned above. When a clinician wishes to *attest* to the state of a workspace, they click a *save* or *attest* button to indicate this. Similar to signing a note, attestation is designed to be used for final versions of documentation that will go into the medicolegal record. Attestation creates a locked past state that can no longer be edited by anyone but is added to the record of past states.

**Figure 10:**
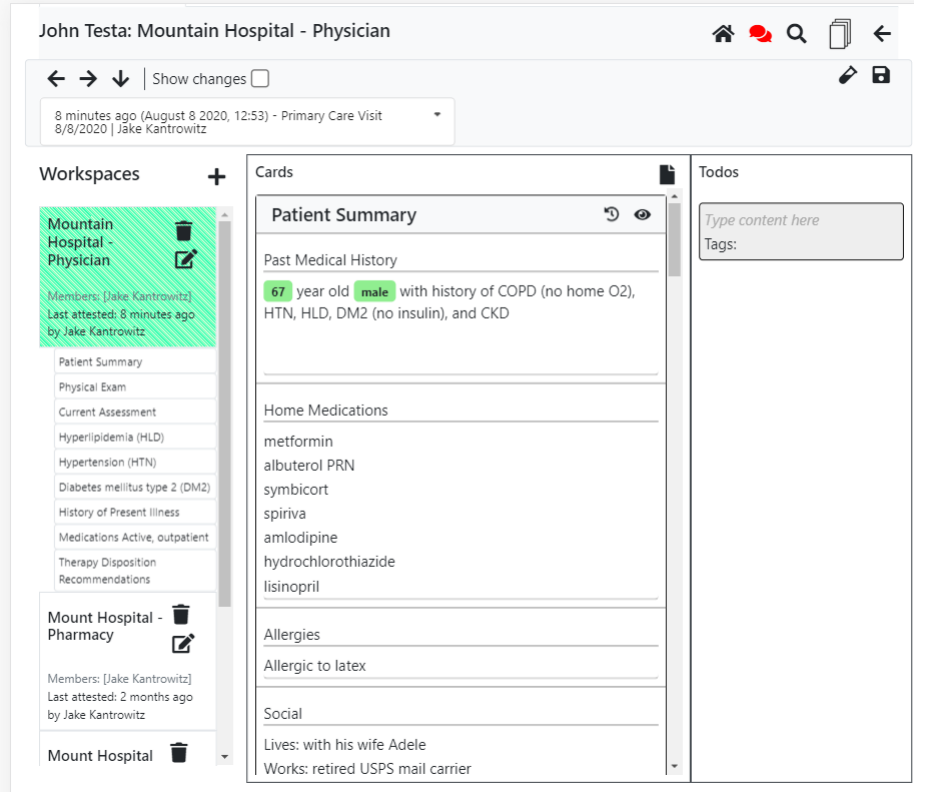
Diagram of the overall organization of a patient interface for a fake patient. Each workspace enables separate clinical teams to keep separate threads on rare occasions when it is necessary. Each workspace stores a history of states; that is, the current state of the workspace and older states of the workspace (past versions). These can be scrolled through easily using the left and right arrows at the top.

**Figure 11:**
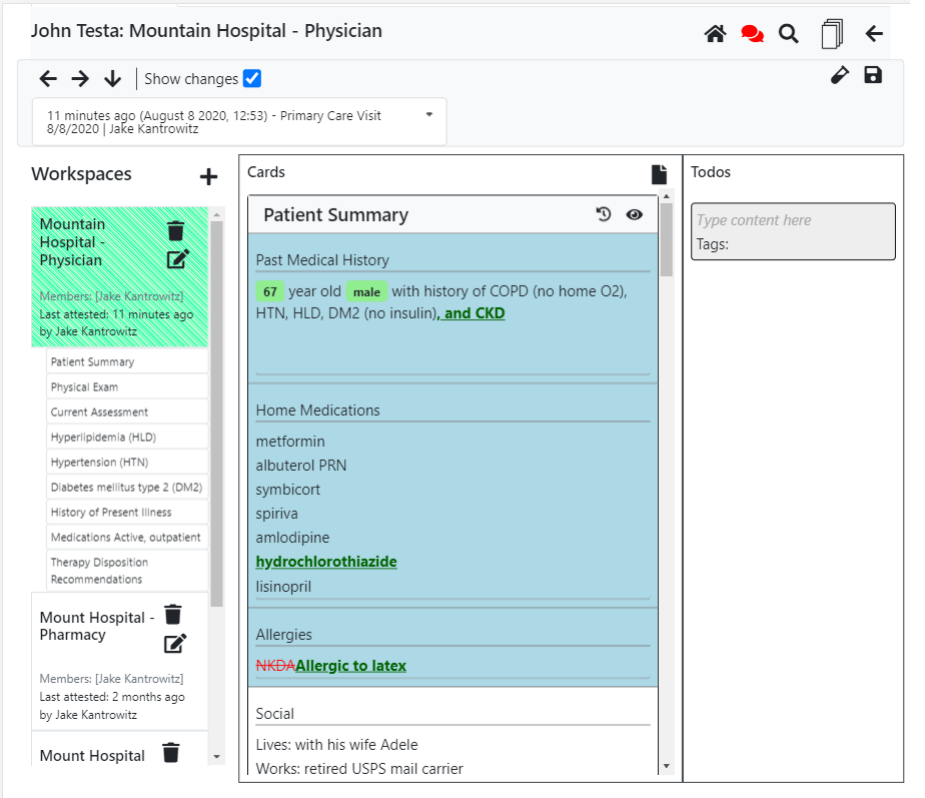
When the show changes button is checked, a track-changes view occurs, enabling later readers to quickly understand what has changed from one state to the next (for instance, the addition of a new diagnosis, medication, and allergy).

Users can scroll back and forth through previous workspace states to quickly see how information has changed over time (Figure 10). A prominent *show changes* button enables intuitive highlighting of cards, fields, and individual sentences (Figure 11), enabling quick focus on the small pieces of information that have changed from state *t* to state *t+1*. Although nothing in principle prevents a version history system from being implemented in note-based EMRs, our card-based organization maximizes its use by enabling individual cards or fields to be tracked over time. Software should be optimized for the most common use cases; tracking medical problems over time is perhaps the single most common use case of an EMR, but the *note* abstraction is ill-suited for it.

Clinicians can also export the current state of a workspace to a single block of text, enabling our system to be compatible and interoperable with note-based systems. For instance, clinicians could document in our system and export a workspace state as a note-like block of text to another documentation system. This would enable our system to be used as an adjunct to a note-based EMR and facilitate direct comparative studies of usability without requiring large-scale institutional changes.

### Team View

Another major source of information scatter rests at the level of the *team* or *patient group*. An extremely common EMR use case involves clinicians managing groups of patients (eg, an inpatient team or an outpatient panel) and either (1) looking for updates in the state of any patient in the group or (2) *running the list*—identifying the status and action items of a group of patients in rapid sequence. Team management and *list-running* are core features of our software. The *team view* interface aggregates the latest state of all workspaces for all patients on a particular team, enabling highly granular actions to be taken for individual patients from a single screen without any requirement to navigate between charts. The team view displays the list of cards for each workspace, enabling quick addition or editing of specific card fields with new single pieces of information. This enables immediate placement of information in topic-appropriate locations—a feature designed to facilitate keeping the chart organized, preventing the need to redocument the same information in different places or scatter information across multiple locations. In addition, the to-do items for each workspace are aggregated and viewable on one page, enabling efficient list running even for large groups of patients.

## Discussion

### Principal Findings

We demonstrated the feasibility of building an EMR that does not use the *note* as the core organizational unit for unstructured data. This is made possible by reimagining the chart as a fully collaborative, dynamic workspace, with information organized primarily by topic rather than by time or thread. This paradigm shift facilitates the use of a wide variety of strategies and design elements that have the potential to reduce information overload and information scatter, two of the key pathologies of modern electronic documentation. Our system is designed to accomplish this goal by disincentivizing the behaviors that lead to these pathologies, such as redundant documentation over time and across multiple threads. We believe that this type of EMR data organization has the potential to reduce clinician documentation time; increase direct face-to-face time with patients; mitigate medical errors resulting from conflicting or erroneous information; create cleaner, more intuitive patient charts; and improve clinician satisfaction with the EMR. We also believe that our organizational paradigm is more well-suited to the actual practice of medicine in the 21st century, with its assumptions of team-based medicine, granular data updates (eg, from SMS text messages, phone calls, biosensors, or digital health devices), and large amounts of information to manage and organize. We hope this study can begin a conversation among clinicians and institutions about the pros and cons of using *notes* as the primary organizational paradigm going forward.

In addition, our system casts doubt on traditional delineations of *structured data* and *unstructured data* by enabling individual clinicians to create their own structured data elements for customized workflows. This decision is motivated by the desire to enable maximal customization for common workflows, such as the workup of common presentations. Too little structure (eg, undifferentiated blocks of free text or loosely templated notes) limits the potential for efficiency gains through automated or stereotyped documentation workflows, whereas too much structure (eg, entering all data in the form of checkboxes, radio buttons, and drop-down menus) is time consuming and implausible for narrative information such as patient histories and clinician thought processes. Rather than being limited by historical decisions about what *structured data* should be (eg, meaningful use requirements or EMR certification guidelines), our system empowers clinicians, teams, or institutions to make their own decisions about how to maximize the gains and minimize the costs of documenting in a structured format.

Our study is a logical extension of the dialog around the problem-oriented medical record, which similarly organizes information primarily by topic. However, we expand the topic-based organization to cover the entirety of medical data in the chart and not just the data that can be represented by *medical problems* or *diagnoses*. In addition, we built the assumptions of full editability and collaboration into the core of the system design. Fully collaborative documentation systems have previously been discussed in different contexts [27], and many studies have pointed out how EMR design paradigms can facilitate or block collaboration between clinicians [28,29]. Our work builds on these discussions, and we believe that fully collaborative documentation systems are a key step in reducing documentation burden and information overload.

We have piloted our system with small user groups on mock patient records but have not yet tested it at scale. In addition, to empirically evaluate whether our system succeeds at its goal of reducing information chaos, direct comparisons between note-based paradigms and non–note-based paradigms will be necessary. This will require further development of EMR usability evaluation frameworks, including a standardized set of metrics for comparing EMRs on relevant end points (including efficiency, cognitive load, and time spent documenting). In future work, we aim to develop such a framework and use it to compare our system with other EMR systems. Another key step in this process is the development of open-source standardized data sets with dummy patient records, designed to facilitate head-to-head comparison of systems at particular common clinical tasks, such as information retrieval, chart summarization, various granularities of data entry, or clinical communication. One could imagine a publicly available EMR "obstacle course" with metrics to quantify the performance of an EMR at common clinical tasks and would enable standardized comparisons for clinicians and institutions looking to reduce EMR time. Such a system could be used not only to evaluate the impact of simple interface changes or feature additions but also to quantify the impact of different organizational paradigms.

Full-fledged EMRs are more than mere documentation interfaces and include functionality to place orders, prescribe medications, and perform population health analyses. These functionalities were not the focus of this study, as we focused primarily on information input and retrieval, as well as task management and collaboration. We believe that non-note interfaces open new possibilities for thinking about order entry, decision support, and other core EMR tasks, as well as how to integrate these tasks with *documentation* per se. In the interim, clinicians could also use such systems as improved *documentation assembly* interfaces in parallel with existing EMRs. In our system, individual workspace states can be exported as raw-text *notes* compatible with EMRs that operate under note-based paradigms. Our system can thus be operationalized either as part of a new EMR or alongside existing EMRs as a separate documentation assembly and information retrieval interface. Ultimately, we hope that clinicians, health systems, and technology vendors will consider the benefits of building and deploying EMRs that operate entirely using collaborative, dynamic, and problem-oriented documentation paradigms.

## Appendix

Multimedia Appendix 1 Information chaos.

Multimedia Appendix 2 Technical details.

## Abbreviations

CDS: clinical decision support
CHF: congestive heart failure
EMR: electronic medical record
RIME: reporter, interpreter, manager, and educator
