# Supplementary material for: A Fully Collaborative, Noteless Electronic Medical Record Designed to Minimize Information Chaos: Software Design and Feasibility Study
Source: JMIR Form Res. 2021 Nov 9;5(11):e23789. doi: 10.2196/23789 (PMC8663541; doi:10.2196/23789)
Supplement: Multimedia Appendix 1 [file formative_v5i11e23789_app1.docx]

**Appendix 1: Information chaos**

The “information chaos” framework is useful for understanding the sources of suboptimal documentation and information retrieval practices within medicine. The first source of information chaos is information overload: the phenomenon of “too much information”, which makes it difficult or time-consuming to identify and summarize the relevant clinical data. Information overload often takes the form of *duplicate* or *copy-pasted* information - redundant data documented multiple times by the same clinician, or by different clinicians who document the same information in different places. These *unlinked* copies of duplicate information also make errors difficult to correct, because *every* copy of incorrect information must be changed in order to truly expunge an error from the chart. Second, information underload: when necessary information is not present or inaccessible within the chart. Third, information scatter: when related information, which should be documented in one place, is instead documented in multiple different locations. This makes retrieval and summarization needlessly time-consuming and repetitive, and may lead to incomplete chart searches and subsequent medical errors. Fourth, conflicting information: when multiple sources in the chart disagree about an observation or decision. Some conflict is inevitable in team-based medical practice, where reasonable clinicians may disagree, but other information conflict is due to erroneous transcription or mistaken information. Regardless of the source, if the resolution of the conflict is not clear, it will lead to wasted summarization time and potential medical error if a later reader acts on incorrect information. Fifth, erroneous information: data which is outright *wrong* which, nevertheless, persists in the chart. In systems without a pure “editing” functionality, truly correcting errors is difficult. Later readers may have to search through many notes to identify what the “truth” is - for instance, if one clinician documents a “left-sided deep vein thrombosis” instead of “right-sided deep vein thrombosis” and other clinicians carry forward this mistake.

These forms of information chaos reinforce each other in overt and subtle ways. In many modern systems, it is difficult or time-consuming to perform an effective chart search (e.g., summarizing the time-course of multiple medical problems for a single patient). Clinicians may attempt to “solve” this problem by packing more information into each single *note* in order to prevent having to repeat the onerous work of chart search (in effect, using single *notes* to serve the function of the entire *chart).* While this may be effective in the short-term, in the long term it worsens the information *overload* problem; multiple copies of redundant information proliferate in the chart, making chart search even *more* difficult and true error correction nearly impossible. A user-friendly electronic medical record, then, should specifically disincentivize documentation behaviors which contribute to these forms of information chaos, in order to mitigate information chaos and facilitate quick summarization of patient information by later readers. By contrast, the design principles of ineffective systems will *allow* or even *encourage* these behaviors. In this study, we attempt to build an EMR system explicitly based on this single design principle - to disincentivize and mitigate information chaos in the chart.
