# Supplementary material for: A Fully Collaborative, Noteless Electronic Medical Record Designed to Minimize Information Chaos: Software Design and Feasibility Study
Source: JMIR Form Res. 2021 Nov 9;5(11):e23789. doi: 10.2196/23789 (PMC8663541; doi:10.2196/23789)
Supplement: Multimedia Appendix 2 [file formative_v5i11e23789_app2.docx]

**Appendix 2. Technical details**

Real-time Collaboration

Research on real-time collaborative software systems has a long history, and many data structures and algorithms can be used to implement such systems, including models such as operational transforms (OTs) and conflict-free replicated data types (CRDTs). Software design challenges of real-time collaboration include: how to propagate changes from an editing client to other clients, how to handle simultaneous conflicting changes (e.g. to the same section of text), and how to represent others’ users activity in real time. There has been some recent work studying the advantages of fully *local-first* software design for collaborative applications, and we briefly considered this approach [^26^](https://paperpile.com/c/PZafXu/keOm). However, for the foreseeable future, organizational requirements in healthcare are likely to necessitate traditional server-client architecture. Under this model, changes are executed on the client side and pushed to a centralized server, which then propagates those changes to the other clients.

Our system works as follows: in the back-end database, each text field (card title, field title, field content, task text) is treated as an atomic data element, which can be edited or deleted independently. A buffer of individual data transactions (any action including creating, deleting, editing, or reordering cards, card fields, or tasks) is maintained on the client side, and pushed to the server whenever a new transaction is added. The server is the authoritative source of truth on whether a transaction is successful or not. For instance, a user may try to edit or delete a resource which was already recently deleted by another user; in this case, the server returns a value stating that the action was unsuccessful, and the “incorrect” client receives a fresh copy of the up-to-date workspace. In cases where multiple users attempt to edit the same resource, a simple “last-writer-wins” strategy is used, with the server adjudicating relative order of transaction arrival. In order to mitigate such conflicts on the front end, we show users the cursor positions of other clients and highlight text fields that are currently being edited by another user within a particular workspace. These are standard features in modern collaborative applications that will naturally decrease the frequency of conflicting actions.

Web socket connections are initiated upon entry to a particular workspace or team view, using the SocketIO framework. When the server validates or confirms a transaction by a client, it uses the socket connection to push the most up-to-date state of the workspace to all clients active in that workspace. This facilitates a fast real-time feel, necessary for collaborative applications, to the software.
